# Effect of Ulinastatin on Early Postoperative Cognitive Dysfunction in Elderly Patients Undergoing Surgery: A Systemic Review and Meta-Analysis

**DOI:** 10.3389/fnins.2021.618589

**Published:** 2021-06-21

**Authors:** Mei Duan, Fangyan Liu, Huiqun Fu, Shuai Feng, Xue Wang, Tianlong Wang

**Affiliations:** ^1^Department of Anesthesiology, Xuanwu Hospital, Capital Medical University, Beijing, China; ^2^Department of Library, Xuanwu Hospital, Capital Medical University, Beijing, China

**Keywords:** elderly patients, surgery, perioperative inflammation, postoperative cognitive dysfunction, ulinastatin, meta-analysis

## Abstract

**Background:** Postoperative cognitive dysfunction (POCD) is associated with neuroinflammation by triggering the systemic inflammatory responses. Related studies have demonstrated that ulinastatin, which is a urinary trypsin inhibitor, inhibited the release of inflammatory mediators and improved postoperative cognitive function in elderly patients undergoing major surgery. However, there are controversial results put forwarded by some studies. This systemic review aimed to evaluate the effect of ulinastatin on POCD in elderly patients undergoing surgery.

**Methods:** We searched PubMed, Embase, Cochrane Library, Web of Science, and Ovid to find relevant randomized controlled trials (RCTs) of ulinastatin on POCD in elderly patients undergoing surgery. The primary outcomes included the incidence of POCD and the Mini-Mental State Examination (MMSE) scores. The secondary outcome was the levels of inflammatory cytokines such as tumor necrosis factor (TNF)-α, S100β, C-reactive protein (CRP), interleukin (IL)-6, and IL-10. RevMan 5.3 was used to conduct the meta-analysis.

**Results:** Ten RCTs were included finally. Compared with controls, ulinastatin significantly reduced the incidence of POCD [risk ratio (RR) = 0.29, 95% CI 0.21–0.41, test of RR = 1: *Z* = 7.05, *p* < 0.00001]. In addition, patients in the ulinastatin group have lower levels of TNF-α, S100β, CRP, and IL-6 and higher level of IL-10 in serum following surgery.

**Conclusion:** These findings suggested that ulinastatin can be used as an anti-inflammatory drug for POCD prevention in elderly patients undergoing surgery.

**Systematic Review Registration Number:** CRD42019137449.

## Introduction

Postoperative cognitive dysfunction (POCD) is a central nervous system complication that occurs after surgery in the elderly and is characterized by mental confusion, anxiety, personality changes, and memory impairment. Elderly patients are at high risk of POCD. The occurrence of POCD is correlated with decreased quality of life and increased mortality, and there is a possibility of increased risk for developing dementia, such as Alzheimer's disease (AD) (Bekker et al., 2010; Browndyke et al., 2013; Berger et al., 2014).

Although the mechanism of POCD remains unclear, systemic inflammation and neuroinflammation are regarded as important pathologic processes of POCD (Berger et al., 2014, 2015). Moreover, inhibition of systemic inflammatory response during the early postoperative period improves postoperative cognitive function, reducing the incidence of POCD in the elderly (Zhang et al., 2018). Alarmins were released from the surgical trauma tissue injury or secreted by stimulated leukocytes and epithelial cells, such as high-mobility histone 1, neutrophils, and monocyte cytoplasmic proteins S100A8 and S100A9, as well as systemic endotoxemia, which in turn activate the inflammatory pathway, leading to the release of pro-inflammatory cytokines and anti-inflammatory cytokines, such as tumor necrosis factor (TNF)-α, interleukin (IL)-1β, IL-6, IL-10, and so on (Chan et al., 2012; Schietroma et al., 2013, 2016). After that, the peripheral inflammatory cytokines activate and compromise with the blood–brain barrier (BBB) integrity, allowing increased infiltration of inflammatory factors and macrophages into the brain (Fu et al., 2014), ultimately leading to the damage of neurological function (Leslie, 2017; Wei et al., 2019).

Ulinastatin is a urinary trypsin inhibitor (UTI) that is extracted from human urine, which subsequently can inhibit the enzyme activity and stable lysosomal membrane and effectively reduce the systemic inflammatory response (Atal and Atal, 2016). UTI is widely used in patients with pancreatitis, septicopyemia, disseminated intravascular coagulation, and shock (Inoue et al., 2009). And ulinastatin could inhibit the systemic inflammatory response by directly suppressing the activation of neutrophils and monocyte-macrophages and capture lipopolysaccharide (LPS) and bind to LPS receptors, further inhibiting the LPS-induced systemic inflammatory response (Ma et al., 2016; Li X. F. et al., 2018).

Therefore, it has been speculated that the occurrence of POCD can be reduced by inhibiting inflammation and the release of plasma LPS and directly protecting the BBB after infusing ulinastatin. However, some studies suggested that preoperative prophylactic ulinastatin showed no improvement in the POCD. The discrepancy in these results might be due to the surgical stimuli experienced and the timing and dose of ulinastatin used.

To clarify these, a meta-analysis was conducted to examine the efficacy of ulinastatin for the prevention of early POCD in elderly patients, which assists in the future clinical decision-making process.

## Materials and Methods

**Figure 1:**
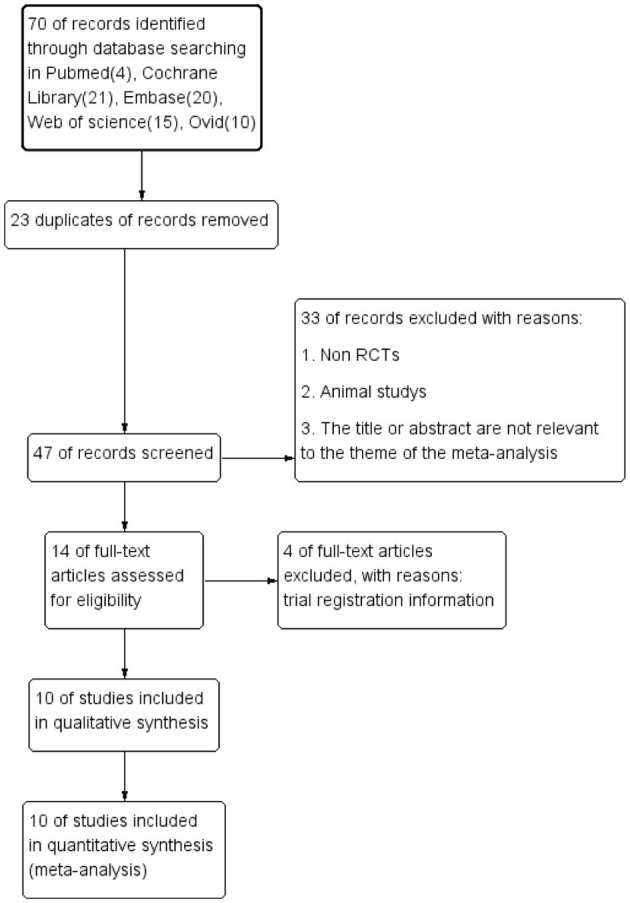
Preferred Reporting Items for Systematic Reviews and Meta-Analyses (PRISMA) diagram of study selection.

Preferred Reporting Items for Systematic Reviews and Meta-Analyses (PRISMA) guidelines were followed in reporting this systematic review and meta-analysis (Figure 1) (Moher et al., 2009a). A review protocol was developed prior to conducting the study.

### Inclusion and Exclusion Criteria

The eligible studies were adopted into this systematic review and meta-analysis following the patient, intervention, comparison, outcomes, and study design strategy (Moher et al., 2009b).

#### Types of Studies

Inclusion: Only randomized controlled trials (RCTs) were included. Exclusion: Observational cohort and case-control studies, case reports, experimental studies, and reviews were excluded.

#### Types of Participants

Subjects enrolled in our systematic review were elderly patients (age ≥60 years) who were undergoing surgical operations, with no restriction of race and gender.

#### Types of Interventions

Patients in the ulinastatin group were treated with ulinastatin intravenously before and/or after surgery; the control group received placebo administration of normal saline, and the volume of normal saline must be the same as that of the administered ulinastatin.

#### Types of Outcome Measurement

The primary outcome measures included the incidence of POCD and the Mini-Mental State Examination (MMSE) score. And the secondary outcome measures were the level of TNF-α, S100β, C-reactive protein (CRP), IL-6, and IL-10 in the blood.

### Search Strategy and Study Selection

We searched PubMed, Embase, Cochrane Library, Web of Science, and Ovid to find relevant RCTs. The search strategy was drafted by an experienced librarian and searched by a combination of medical subject headings and free words. The explicit search strategy used herein was presented in Supplementary File 1. In addition, the literature data that identified the registration test but not published were also searched. The test data were obtained by contacting the appropriate author if necessary.

In addition, each study should have the research site, publication year, and a clear definition of the sample size. At least one outcome indicator, such as odds ratio (OR) or risk ratio (RR), and its 95% confidence interval (CI) should be provided by the study. Detailed description of the intervention group and the control group as well as POCD diagnostic criteria should be clearly stated.

Exclusion criteria were as follows: duplicate articles, review articles, animal experiments, and articles that did not meet the research objectives. Furthermore, the articles that have design defects and poor quality, with incomplete data and unclear outcome, and that cannot provide and convert into OR or RR and its 95% CI, and whose statistical method is incorrect and cannot be corrected were excluded from the meta-analysis.

### Literature Screening, Data Extraction, and Quality Assessment

**Figure 2:**
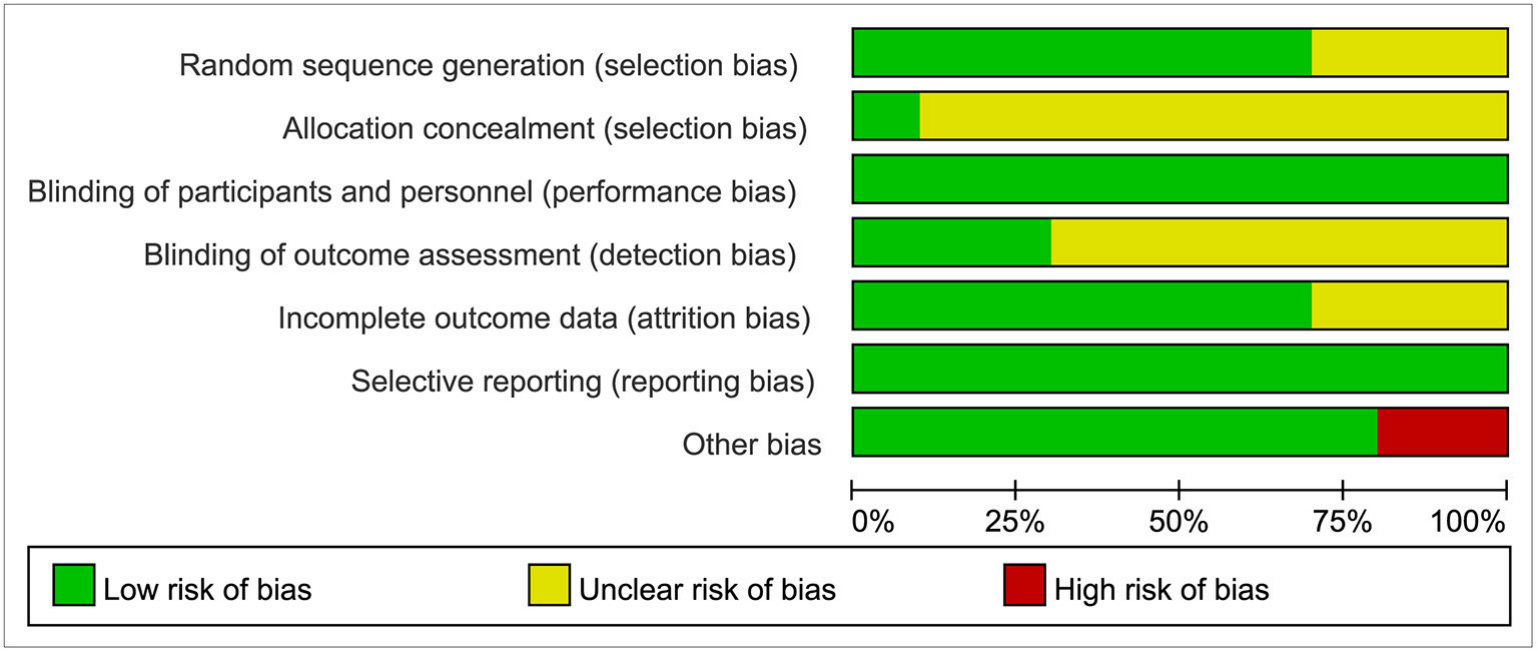
Risk of bias graph: review authors' judgments about each risk of bias item presented as percentages across all included studies.

Two researchers independently screened the literature, extracted the data, and evaluated the methodological quality of the studies identified. We evaluated the methodological quality of 10 RCTs identified by Cochrane risk bias assessment (Higgins et al., 2011). The evaluation contents included random sequence generation, allocation concealment, blinding of investigators and participants, blinding of outcome assessment, incomplete outcome data, selective reporting, and other biases. Any disagreements between the two researchers were solved by discussion or by consulting a third party to reach a consensus. Table 1 showed the extracted contents from 10 RCTs, and the outcome of quality assessment was presented in Figure 2.

**Table 1:** Characteristic of the included studies.

**Study**	**Study design**	**Population**	**Intervention and control**	**Outcomes**
Ge et al. (2011) China	RCT	ASA I–II, age ≥65 and ≤83 years, hip joint replacement under combined spinal-epidural anesthesia U: 80 patients, 72.8 ± 7.5 C: 80 patients, 75.0 ± 9.2	U: intravenous injection of ulinastatin 10,000 U/kg before skin incision, and 5,000 U/kg after surgery on days 1–3 C: an equivalent amount of normal saline	Incidence of POCD
Ge et al. (2015) China	RCT	ASA I–III, age ≥60 and ≤75 years, coronary artery bypass grafting surgery under general anesthesia U1: 31 patients, 69.1 ± 4.8 U2: 30 patients, 68.9 ± 4.7 C: 32 patients, 67.7 ± 5.4	U1 and U2: the 16,000 U/kg or 8,000 U/kg ulinastatin injection was diluted to 60 ml with 0.59% sodium chloride solution, and intravenous infusion was continued at a rate of 20 ml/h before induction of anesthesia C: The control group was given the same volume of 0.59% chlorine by the same method	Incidence of POCD, IL-6, IL-10, TNF-α, S100β
Kang et al. (2010) China	RCT	ASA I–II, age ≥65 and ≤83 years, hip joint replacement under combined spinal-epidural anesthesia U: 40 patients, 75.0 ± 7.8 C: 40 patients, 72.8 ± 7.3	U: intravenous injection of ulinastatin with 10,000 U/kg before skin incision, and 5,000 U/kg after surgery on days 1–3 C: an equivalent amount of normal saline	Incidence of POCD, S100β
Li et al. (2016) China	RCT	ASA II–III, CPB valvular replacement surgery under general anesthesia U: 30 patients C: 30 patients	U: the observation group was pumped into 12,000 U/kg UTI through vein after anesthesia induction, and given 6,000 U/kg UTI from vitro pipeline 5 rain before the end of CPB C: the control group was given the same amount of saline solution	Incidence of POCD, IL-6, IL-10, TNF-α, S100β, NE, SOD, MDA
Pan et al. (2016) China	RCT	ASA I–II, age ≥65 and ≤85 years, laparoscopic colorectal cancer surgery under general anesthesia U: 41 patients, 72.4 ± 7.5 C: 41 patients, 73.9 ± 8.4	U: 2 KU/kg ulinastatin before induction of anesthesia, followed by 1 KU/(kg·h) intravenous pumping until the end of surgery C: the same volume of normal saline	Incidence of POCD, MMSE score on the day of surgery and postoperative day 1/3, TNF-α, IL-6, TGF-β, IL-4
Shan et al. (2015) China	RCT	ASA I–II, age ≥65 years, hip fracture surgery under combined spinal-epidural anesthesia U: 21 patients, 78.0 ± 2.0 C: 27 patients, 75.0 ± 1.0	U: intravenous injection of ulinastatin with 5 000 U/kg before skin incision and at the moment of the end of operation C: the same volume of normal saline	Incidence of POCD, MMSE score on the day of surgery and postoperative day 1/3/7, CRP
Wang et al. (2017) China	RCT	ASA I–II, age ≥60 years, TNM II–III patients after one lung ventilation surgery under intravenous general anesthesia and receiving neoadjuvant chemotherapy U: 38 patients, 66.0 ± 6.0 C: 37 patients, 67.0 ± 5.0	U: ulinastatin 10,000 U/kg diluted to 100 ml with normal saline and infused intravenously over a period of 20 min before anesthesia induction and 5,000 U/kg after surgery on days 1–3 C: an equivalent amount of normal saline	Incidence of POCD, MMSE score on postoperative day 7, IL-6, IL-10, CRP, S100β
Lili et al. (2013) China	Double-blind trial RCT	ASA I–II, age ≥65 years, abdominal surgery under intravenous general anesthesia U: 40 patients, 75.6 ± 7.2 C: 40 patients, 74.1 ± 8.1	U: ulinastatin 10,000 U/kg diluted to 100 ml with normal saline and infused intravenously over a period of 30 min before surgical incision and 5,000 U/kg after surgery on days 1–3 C: an equivalent amount of normal saline	Incidence of POCD, IL-6, TNF-α, CRP, S100β
Yang et al. (2017) China	RCT	ASA I–II, age ≥65 years, laparoscopic gastrectomy surgery under general anesthesia U: 50 patients, 70.6 ± 6.7 C: 40 patients, 69.5 ± 5.3	U: patients in the combination group were first injected with ulinastatin 10,000 U/kg within 15–20 min. Then, dexmedetomidine was administered in the same manner as the control group, and the administration was stopped 30 min before the end of the operation C: before the induction of anesthesia, the control group was given a loading dose of dexmedetomidine 0.5 μg/kg, after 15 min of infusion, the pump was continuously pumped at 0.3 μg/kg·h, and the infusion was stopped 30 min before the end of the operation	Incidence of POCD, MMSE score on the day of surgery and postoperative day 1/7, IL-6, S100β, TNF-α
Zhang et al. (2018) China	Double-blind trial RCT	ASA I–II, age ≥65 and ≤85 years, spine surgery under intravenous general anesthesia U: 30 patients, 72.8 ± 5.3 C: 30 patients, 71.3 ± 5.0	U: intravenous infusion of ulinastatin 10,000 U/kg following anesthesia induction and before surgical incision, and 5,000 U/kg on post-operative days 1 and 2 C: an equivalent amount of normal saline	Incidence of POCD, MoCA score on postoperative day 7, IL-6, CRP, LPS, MMP-9

*SOD, Superoxide Dismutase; MDA, Malondialdehyde; MMSE, Mini-Mental State Examination; TGF-β, Transforming Growth Factor-β; MoCA, Montreal Cognitive Assessment; MMP-9, Matrix Metalloprotein-9*.

Grading of Recommendations, Assessment, Development, and Evaluations (GRADE) methods were used to evaluate the quality of evidence for each outcome, classified as very low, low, moderate, or high (Balshem et al., 2011). It was evaluated using GRADEPro software 3.6 (GRADE Working Group).

### Statistical Analysis

We used RevMan 5.3 to conduct this meta-analysis. For dichotomous data, RRs with 95% CIs were used to express the effect sizes, while mean difference (MD) and 95% CIs were used for continuous data.

Firstly, we conducted a heterogeneity test (significance level, α = 0.10) to evaluate the extent of heterogeneity in combination with the *I*^2^-test (Higgins et al., 2003). A fixed-effects model or a random-effects model was selected based on the results of *I*^2^-test. A fixed-effects model was used to conduct the meta-analysis if no heterogeneity (*p* > 0.1 and *I*^2^ < 50%) was observed among the studies. If significant heterogeneity (*p* ≤ 0.1 or *I*^2^ ≥ 50%) was observed, then a random-effects model was used for the meta-analysis. The *Z*-test was used to determine the significance of the pooled effect size, and a *p* < 0.05 was considered statistically significant. In addition, if there was significant heterogeneity among the studies, subgroup or sensitivity analysis should be conducted.

Publication bias was assessed using the funnel plots, Egger's regression test (Egger et al., 1997), and Begg's adjusted rank correlation (Begg and Mazumdar, 1994), which was conducted with the Stata software (Stata Corp., TX, USA; version 15.0).

## Results

### Literature Search Results

Initially, 70 relevant studies were identified in total. Of these, 23 duplicates and 33 records deviating from inclusion criteria were excluded. In addition, four trial registration data were excluded. Finally, 10 RCTs were included in the meta-analysis after reviewing the full text of each article and then were evaluated with the RCT Quality Assessment Scale. The results of the literature screening process according to PRISMA are shown in Figure 1.

### Basic Features and Quality Assessment of Included Studies

The characteristics of the included studies are presented in Table 1. We applied Cochrane risk bias assessment in the quality evaluation of the included studies, and most studies were at low risk of bias as shown in Figure 2.

### Meta-Analysis Results

#### Incidence of Postoperative Cognitive Dysfunction

**Figure 3:**
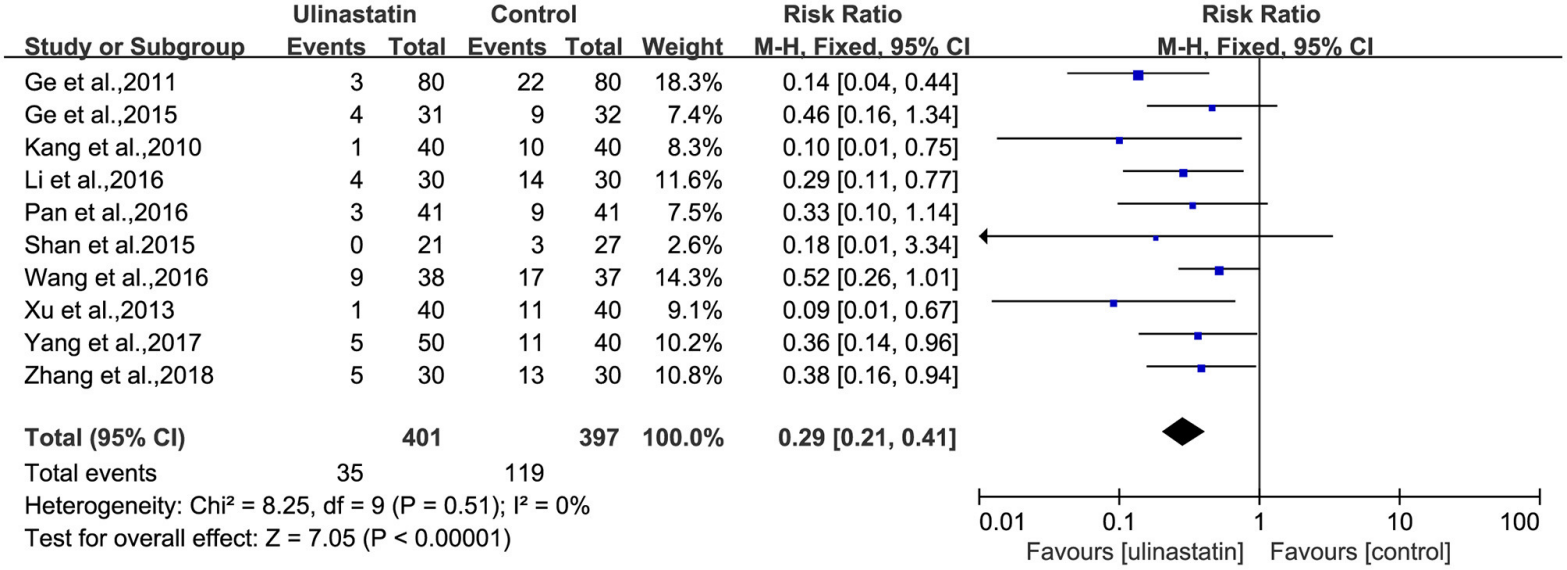
Forest plot: Meta-analysis and pooled risk ratio (RR) of the effect of ulinastatin on postoperative cognitive dysfunction (POCD).

**Figure 4:**
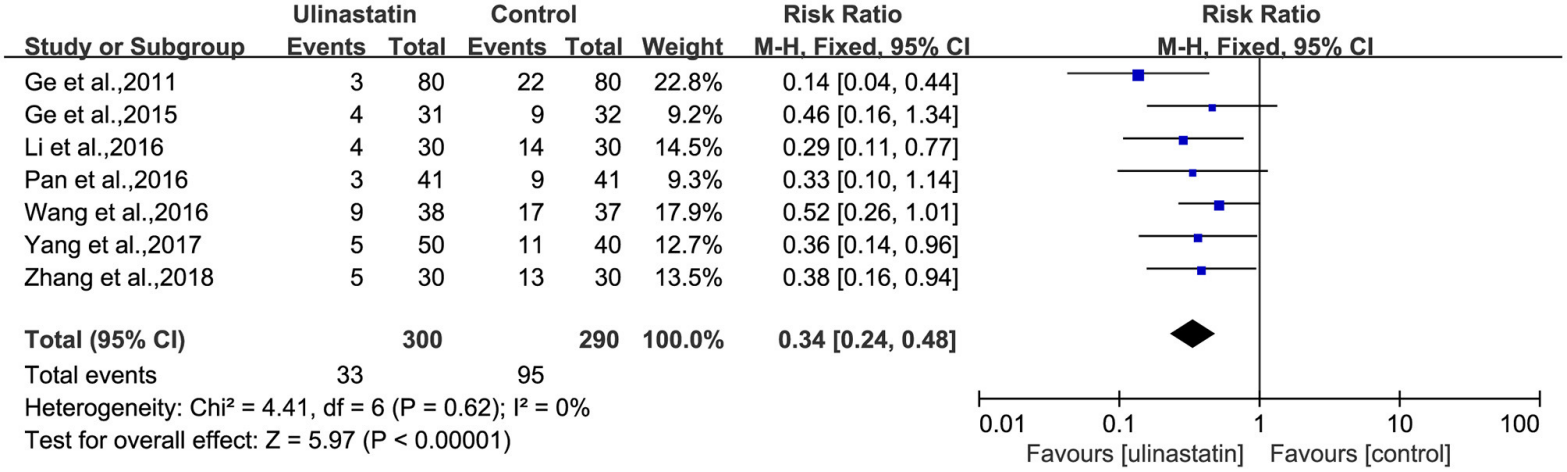
Forest plot: Meta-analysis and pooled risk ratio (RR) of the effect of ulinastatin on postoperative cognitive dysfunction (POCD) after removing three articles with publication biases.

There was no substantial heterogeneity among the studies (*p* = 0.51, *I*^2^ = 0%; Figure 3). Therefore, a fixed-effects model was applied in this meta-analysis, and the results presented by the forest map showed that the incidence of POCD in the ulinastatin group was significantly lower than that in the control group (RR = 0.29, 95% CI 0.21–0.41, test of RR = 1: *Z* = 7.05, *p* < 0.00001; Figure 3). To verify the stability of the effects of the interventions, a sensitivity analysis was conducted where each study was eliminated to determine the variation in results. The results showed that there was no significant difference between the results obtained after the knockout and the total combined values (RR = 0.290, 95% CI 0.206–0.409; Supplementary File 2). The GRADE quality of evidence for POCD rate was judged to be moderate. The funnel plot of POCD was a little asymmetrical (Supplementary File 3). The Egger's regression asymmetry test (*p* = 0.011) and the Begg's adjusted rank correlation test (*p* = 0.049) showed evidence of publication bias regarding POCD rates. Therefore, we removed Xu, Shan, and Kang, which had obvious publication biases. And we analyzed the forest plot for POCD rates again, and the result showed that there was still a lower POCD rate in the ulinastatin group than that that in the control group, the same as before removing (RR = 0.34, 95% CI 0.24–0.48, test of RR = 1: *Z* = 5.97, *p* < 0.00001; Figure 4). The influence analysis of individual studies on the pooled RR is presented that the benefit effect of ulinastatin on POCD is stable (RR = 0.336, 95% CI 0.235–0.481; Supplementary File 4). The GRADE quality of evidence for POCD rate was judged to be high after removing three articles with publication biases. And the funnel plot of POCD after removing the three articles with publication bias was nearly symmetrical (Supplementary File 5). The Egger's regression asymmetry test (*p* = 0.087) and the Begg's adjusted rank correlation test (*p* = 0.133) showed no publication bias regarding POCD rates.

#### Postoperative Mini-Mental State Examination Score

Three RCTs reported the MMSE score on day 1 after surgery. Three RCTs reported the MMSE score on day 3 after surgery, and three RCTs reported the MMSE score on day 7 after surgery.

**Figure 5:**
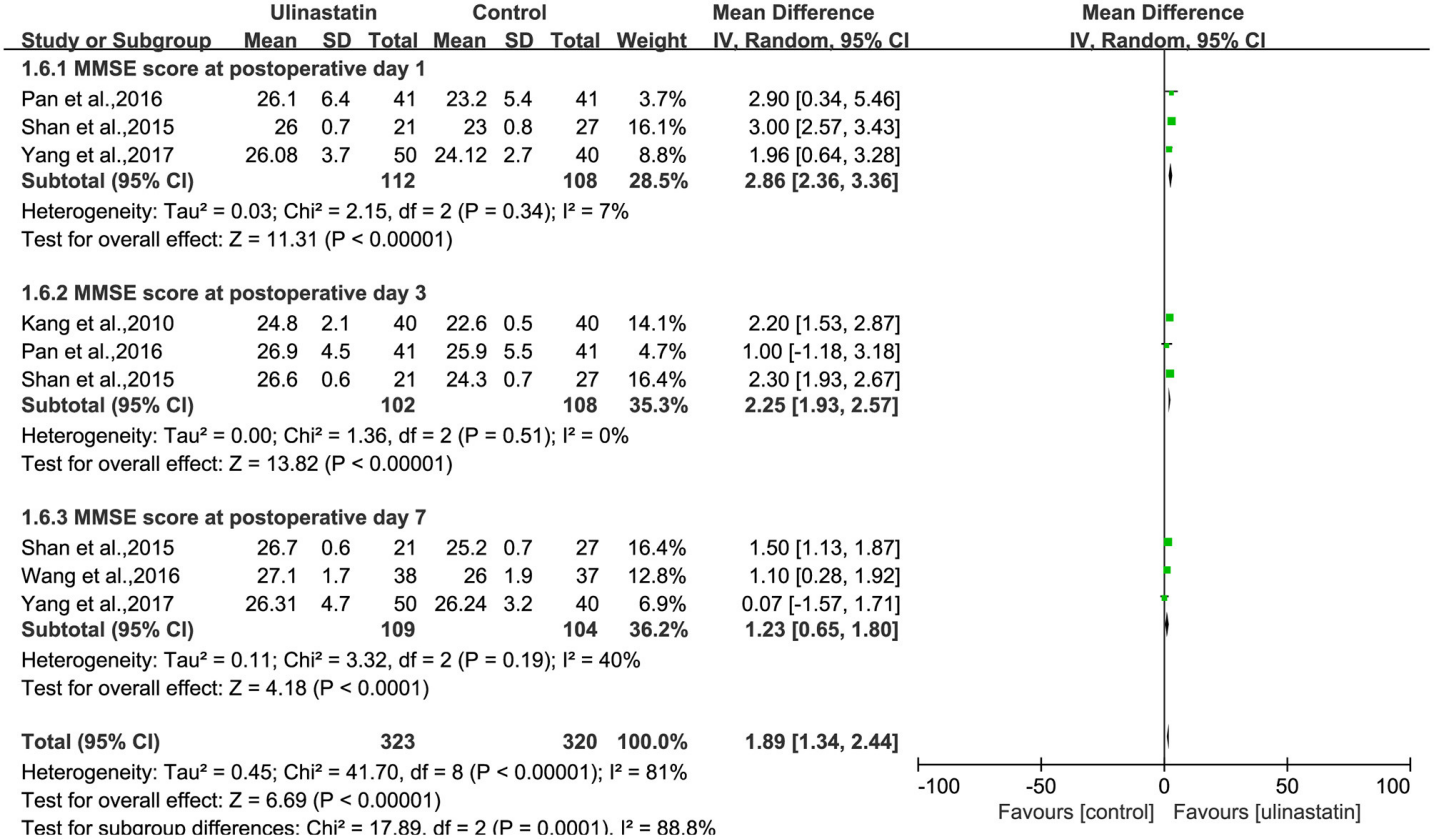
Forest plot: Comparing the Mini-Mental State Examination (MMSE) scores of patients receiving ulinastatin vs. control divided by postoperative days 1, 3, 7.

A substantial heterogeneity was observed among the three subgroups (*p* < 0.00001, *I*^2^ = 81%; Figure 5) and in each group (*p* = 0.34, *p* = 0.51, *p* = 0.19; *I*^2^ = 7%, *I*^2^ = 0%, *I*^2^ = 40%; Figure 5). Then, the random-effects model was used in the meta-analysis, and the outcomes presented showed significant differences about MMSE scores after surgery between the ulinastatin group and the control group. It was obvious that the MMSE score after surgery in the ulinastatin group was higher than that in the control group (MD = 1.89, 95% CI 1.34–2.44, test of RR = 1: *Z* = 6.69, *p* < 0.00001; Figure 5). The GRADE quality of evidence for the MMSE score was judged to be high. The funnel plot of the MMSE score at postoperative day was symmetrical, indicating no publication bias among the 10 RCTs (Supplementary File 6). The Egger's regression asymmetry test (*p* = 0.061, *p* = 0.253, *p* = 0.104) and the Begg's adjusted rank correlation test (*p* = 1.000, *p* = 0.296, *p* = 0.296) showed no publication bias regarding MMSE scores at postoperative days 1, 3, and 7, respectively.

### The Level of Inflammatory Factors

**Figure 6:**
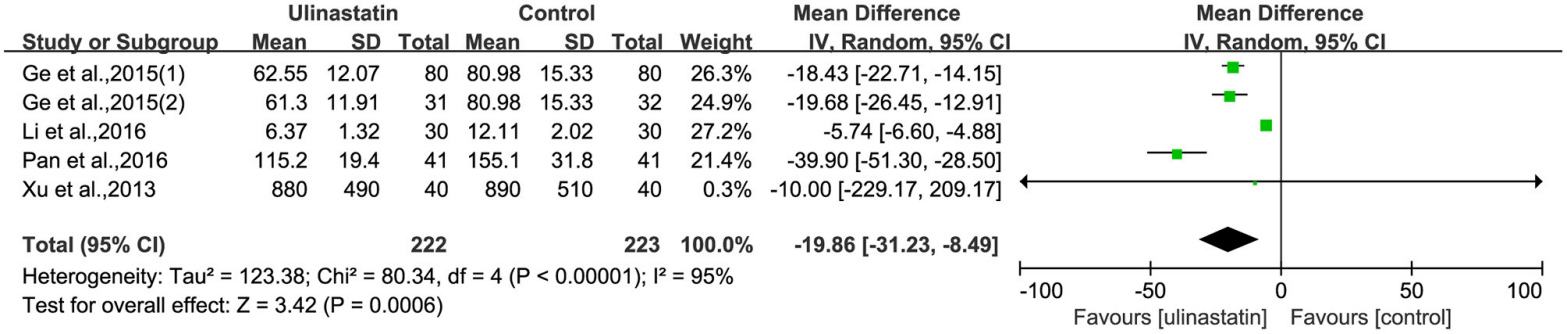
Forest plot: Comparing plasma tumor necrosis factor (TNF)-α of patients receiving ulinastatin vs. control.

**Figure 7:**
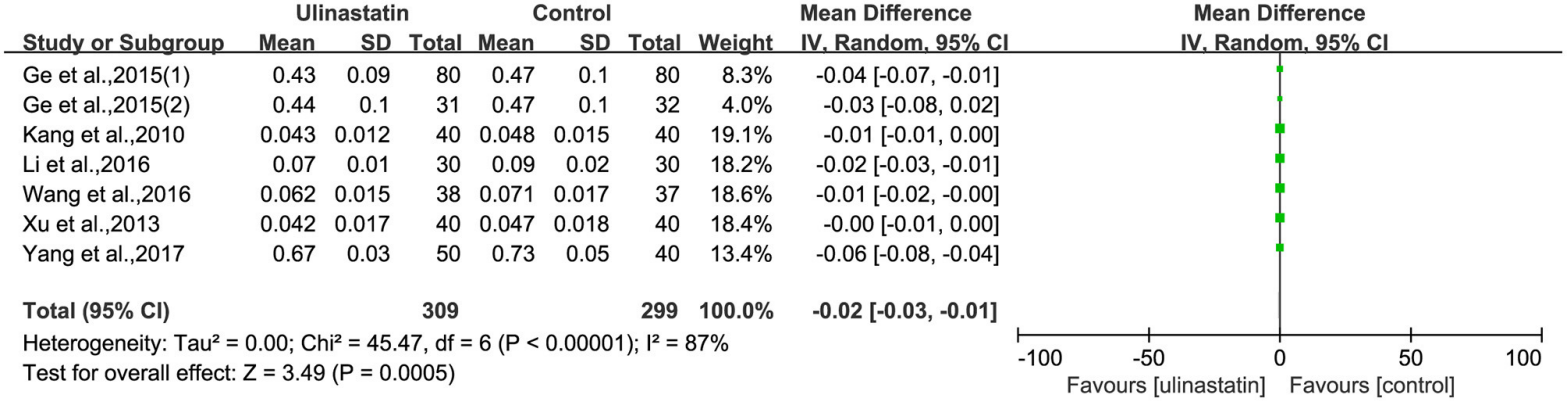
Forest plot: Comparing plasma S100β of patients receiving ulinastatin vs. control.

**Figure 8:**
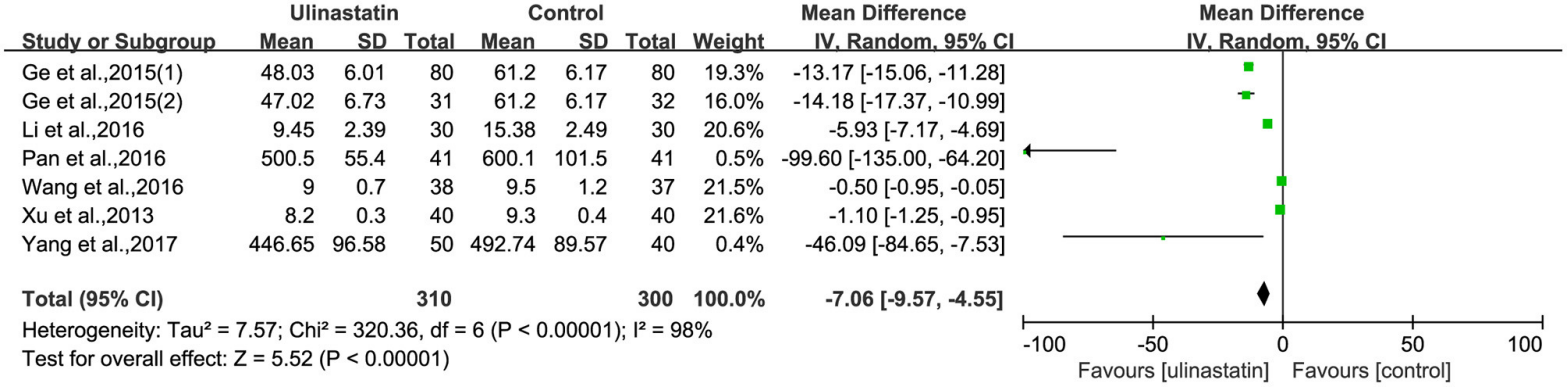
Forest plot: Comparing plasma interleukin (IL)-6 of patients receiving ulinastatin vs. control.

**Figure 9:**
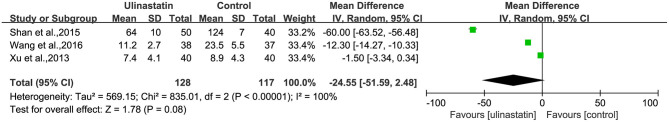
Forest plot: Comparing plasma C-reactive protein (CRP) of patients receiving ulinastatin vs. control.

**Figure 10:**
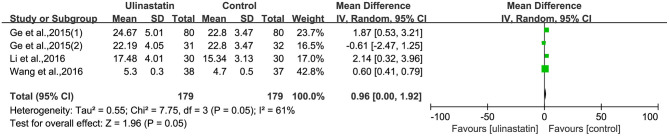
Forest plot: Comparing plasma interleukin (IL)-10 of patients receiving ulinastatin vs. control.

The relevant inflammatory factors were also measured among the 10 RCTs included. From the results of these inflammatory factors, we found no difference in the preoperative inflammatory factor levels between the ulinastatin group and the control group. The levels of TNF-α, S100β, and IL-6 were significantly increased after surgery (Supplementary File 7). Furthermore, the control group had significantly higher levels than those in the ulinastatin group, and the difference was statistically significant (test of RR = 1 : Z = 3.42, 3.49, 5.52, *P* < 0.05; Figures 6–8). Also the CRP levels were elevated postoperatively in the two groups and was higher in the ulinastatin group than that in the control group, but showing no significant difference (test of RR =1: *Z* = 1.78, *p* = 0.08; Figure 9). Inversely, the level of IL-10 was higher postoperatively in the ulinastatin group than that in the control group (test of RR = 1: *Z* = 1.96, *p* = 0.05; Figure 10). In a word, these findings suggest that ulinastatin can attenuate the development of POCD and improve the MMSE scores after surgery, which is most likely through a reduction of TNF-α, S100β protein, pro-inflammatory IL-6, and CRP and an increase of anti-inflammatory IL-10 levels.

## Discussion

This article systematically analyzed the protective effects of ulinastatin in the treatment of patients with early POCD. Ten RCTs were included in this meta-analysis. Based on the findings of this study, ulinastatin effectively reduced the incidence of early POCD by inhibiting the release of pro-inflammatory cytokines and promoting the upregulation of anti-inflammatory cytokine IL-10.

TNF-α and IL-6 are pro-inflammatory cytokines that are associated with cognitive impairment (Liu et al., 2018). A variety of rodent surgical models show that pro-inflammatory cytokines are both upregulated in peripheral tissue and central nervous system (Terrando et al., 2010; Fidalgo et al., 2011). In human studies, the serum and cerebrospinal fluid of patients with postoperative cognitive impairment also showed an increase in pro-inflammatory cytokines, which had nothing to do with the type of operation (Buvanendran et al., 2006; Hirsch et al., 2016), which has been confirmed by a meta-analysis (Peng et al., 2013). Tissue trauma after an operation releases IL-1β and TNF-α and further promotes the increase of IL-6 cytokine, which is related to the degree of tissue trauma (Mannick et al., 2001; Menger and Vollmar, 2004). Pro-inflammatory factor IL-6 is considered to be an independent predictor of postoperative cognitive impairment (Dong et al., 2016), and perioperative inflammatory factors are closely related to the occurrence of postoperative cognitive impairment in elderly patients (Li et al., 2012). Our included studies illustrated that the levels of serum TNF-α and IL-6 were significantly increased after surgery, and ulinastatin may attenuate POCD by inhibiting the release of TNF-α and IL-6.

Moreover, ulinastatin was proven to upregulate anti-inflammatory factor IL-10 (Lili et al., 2013), which was associated with improvement of postoperative cognitive function (Lili et al., 2013; Wang et al., 2017). Ulinastatin can decrease the levels of pro-inflammatory cytokines TNF-α, CRP, and IL-6 by activating phosphoinositide 3-kinase (PI3K)/Akt/Nrf2 pathway and promote the release of anti-inflammatory cytokine IL-10 by inhibiting c-Jun N-terminal kinase (JNK)/nuclear factor (NF)-κB pathway (Li S. T. et al., 2018). Relevant findings demonstrate that ulinastatin can attenuate the elevation of S100β protein levels and the incidence of POCD, most likely by the mechanism of reducing serum IL-6 and CRP levels and increasing IL-10 levels (Lili et al., 2013). These were also consistent with our study.

Though the meta-analysis result of the plasma CRP was not significantly different between the ulinastatin and the control group, possibly on account of less cases, relevant studies showed that the plasma CRP at postoperative day 3 was more likely to promote the occurrence of POCD (Zhang et al., 2015). Hudetz et al. (2011) stated that IL-6 and CRP were significantly elevated in plasma of patients with postoperative memory impairment. The above evidence indicated that systemic inflammation was regarded as an important pathologic process of POCD (Berger et al., 2014, 2015), and ulinastatin plays a critical role in the inhibition of early POCD.

The BBB integrity compromised by the peripheral inflammatory cytokines may allow inflammatory factors and macrophages into the brain directly (Fu et al., 2014) or cause the release of molecules, further reflecting neuronal damage (Thelin et al., 2017). Serum S100β is one of these molecules and is an acidic calcium-binding protein that is first found in astrocytes and Schwann cells (Linstedt et al., 2002). S100β is usually elevated in the blood and cerebrospinal fluid following nervous system damage during BBB impairment, which has been considered as a biomarker of cognitive impairment (Linstedt et al., 2002; Li et al., 2012). Ulinastatin was found lowering the concentration of plasma S100β in the first 2 days after an operation (Kang et al., 2010; Lili et al., 2013). Ulinastatin might downregulate the level of S100β in blood through impeding the inflammatory cascades and lowering the level of pro-inflammatory cytokines TNF-α, CRP, and IL-6 (Wang et al., 2017), further protecting the BBB integrity from peripheral inflammatory cytokines and decreasing neuronal damage. Our included studies showed that the levels of serum S100β were significantly increased after surgery. The concentration of postoperative serum S100β in the ulinastatin group was significantly lower than that in the control group. Furthermore, the incidence of POCD in the ulinastatin group was lower than that in the control group. These results demonstrated that ulinastatin may attenuate POCD by inhibiting the generation of S100β.

Systemic inflammatory response has been proven to be induced by gut microbiome-driving LPS with surgery-induced intestinal barrier dysfunction (Rhee, 2014; Schietroma et al., 2016). According to a trial that was included in our study, the level of serum LPS showed an association with the incidence of POCD (Zhang et al., 2018). LPS protein complex with toll-like receptor-4 activating the cellular NF-κB signaling pathway led to the increase of pro-inflammatory cytokines in the blood and in the brain (Li et al., 2004; Brun et al., 2007). The intraperitoneal injection of LPS triggered systemic inflammation and neuroinflammation, consequently inducing the cognitive function in aged rats (Kan et al., 2016). Zhang et al. (2018) found that the serum LPS and the occurrence of POCD were lower in the ulinastatin group than those in the control group. Studies showed that ulinastatin also inhibited the inflammatory cascade triggered by LPS in the blood. Furthermore, ulinastatin reduced the intestinal protease content, slowed down the digestion of intestinal tissue by protease, and reduced the damage of intestinal mucosa during ischemia, thereby protecting the intestinal barrier function. This article did not cover this, and so more rigorous research is needed to confirm this.

However, Li et al. (2013) suggested that ulinastatin could not improve cognitive function in aged rats. The intensity of systemic inflammatory response varied with the types of surgery, and this might be the reason for the differential effects of ulinastatin on early POCD from various types of surgeries. It was probably insufficient for the inhibition of initiation and degree of inflammatory response. In addition, it might also be related to the underlying inflammatory state and cognition of different age groups. In a word, there are confounding factors that affected the function of ulinastatin, and the dose of ulinastatin administered during the perioperative period remains to be considered.

There are some limitations in this study. Firstly, the lack of gray literature may lead to publication biases. Secondly, the timing and dose of ulinastatin were not consistent in the included studies, possibly affecting the results of the analysis. In addition, it was recommended that the diagnostic criteria for POCD should be aligned with the clinical diagnostic criteria of neurocognitive disorders such as those that have already been used in the Diagnostic and Statistical Manual for Mental Disorders, Fifth Edition (DSM-5). Furthermore, POCD has been defined in previous research studies to describe an objectively measurable decline in cognitive function at varying intervals after anesthesia and surgery, i.e., up to 3–12 months after surgery (Evered et al., 2018). In our study, POCD was diagnosed by MMSE scores in short-term after surgery in the included studies. Therefore, the effect of ulinastatin on surgery-induced POCD should be evaluated according to the recommended diagnostic criteria of POCD from The Nomenclature Consensus Working Group.

In summary, the prophylactic use of ulinastatin can effectively reduce the incidence of early cognitive impairment after surgery in elderly patients by reducing inflammation. However, due to certain limitations of the quality and quantity of the included studies, the results of this study should be considered with more caution, and more high-quality, large-sample RCTs are needed to verify the results.

## Conclusions

This systematic review and meta-analysis of available evidence suggested that the application of ulinastatin reduced the incidence of early POCD and improved the MMSE score by attenuating the elevated serum levels of TNF-α, S100β protein, and IL-6 and increasing serum IL-10 levels. Nevertheless, high-quality RCTs that are adequately powered are needed to address the shortcomings of this study.

## Data Availability Statement

All data generated or analyzed during this study are included in this published article.

## Author Contributions

MD and FL designed and conceived the study, participated in the acquisition, analysis and interpretation of data, and drafted the manuscript. SF and XW participated in the analysis and interpretation of data and contributed to the manuscript. TW and HF conceived the study, participated in its design and coordination, and helped to draft the manuscript. All authors read and approved the final manuscript.

## Conflict of Interest

The authors declare that the research was conducted in the absence of any commercial or financial relationships that could be construed as a potential conflict of interest. The reviewer WO declared a past co-authorship with one of the authors TW to the handling editor.

## Supplementary Material

Supplementary File 1 The search strategy formula about the Meta-Analysis.

Supplementary File 2 Sensitivity analysis graph: evaluating the stability of the effect of ulinastatin on POCD.

Supplementary File 3 Funnel plot about the incidence of POCD among included studies.

Supplementary File 4 Sensitivity analysis graph: evaluating the stability of the effect of ulinastatin on POCD after removing three articles with publication bias.

Supplementary File 5 Funnel plot about the incidence of POCD after removing three articles with publication bias.

Supplementary File 6 Funnel plot about the MMSE score among included studies.

Supplementary File 7 Changes in inflammatory factors reported by included studies.

## Abbreviations

POCD: postoperative cognitive dysfunction
AD: Alzheimer's disease
MMSE score: Mini-Mental State Examination score
UTI: urinary trypsin inhibitor
RCTs: randomized controlled trials
BBB: blood–brain barrier
DSM-5: Diagnostic and Statistical Manual for Mental Disorders, Fifth Edition
RR: related risk
ORs: odds ratios
95% CI: 95% confidence interval
SMD: standard mean difference.
