# Supplementary figures and images for: Effect of Ulinastatin on Early Postoperative Cognitive Dysfunction in Elderly Patients Undergoing Surgery: A Systemic Review and Meta-Analysis
Source: Front Neurosci. 2021 Jun 21;15:618589. doi: 10.3389/fnins.2021.618589 (PMC8265373; doi:10.3389/fnins.2021.618589)

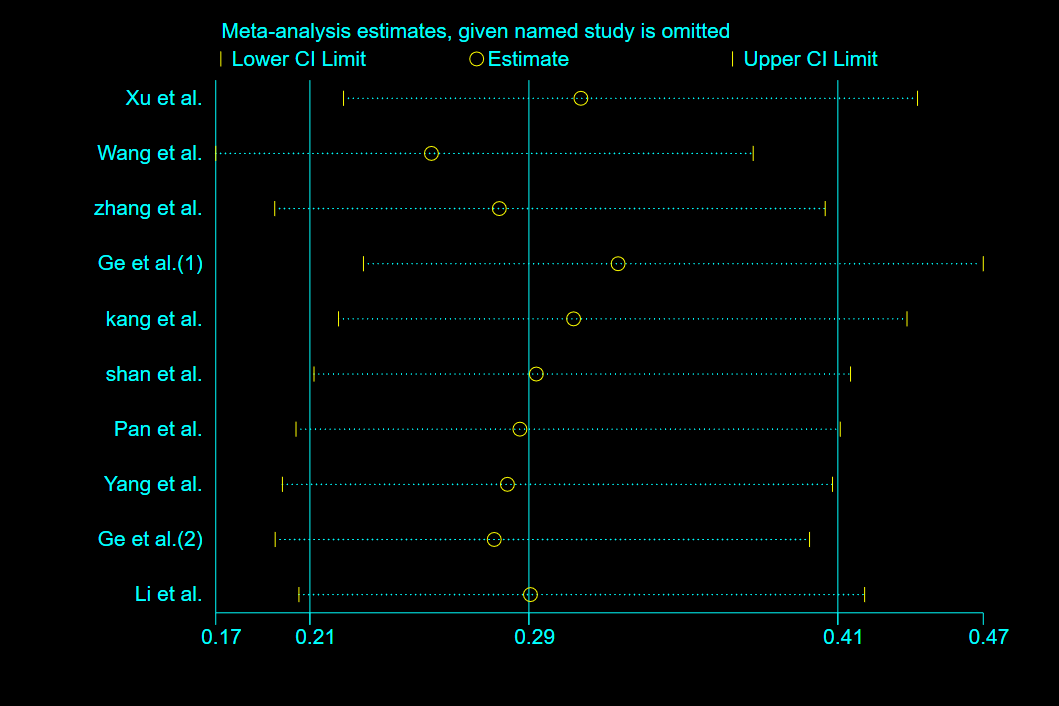

Supplement: Supplementary File 1 — The search strategy formula about the Meta-Analysis. [file Image_1.TIF]

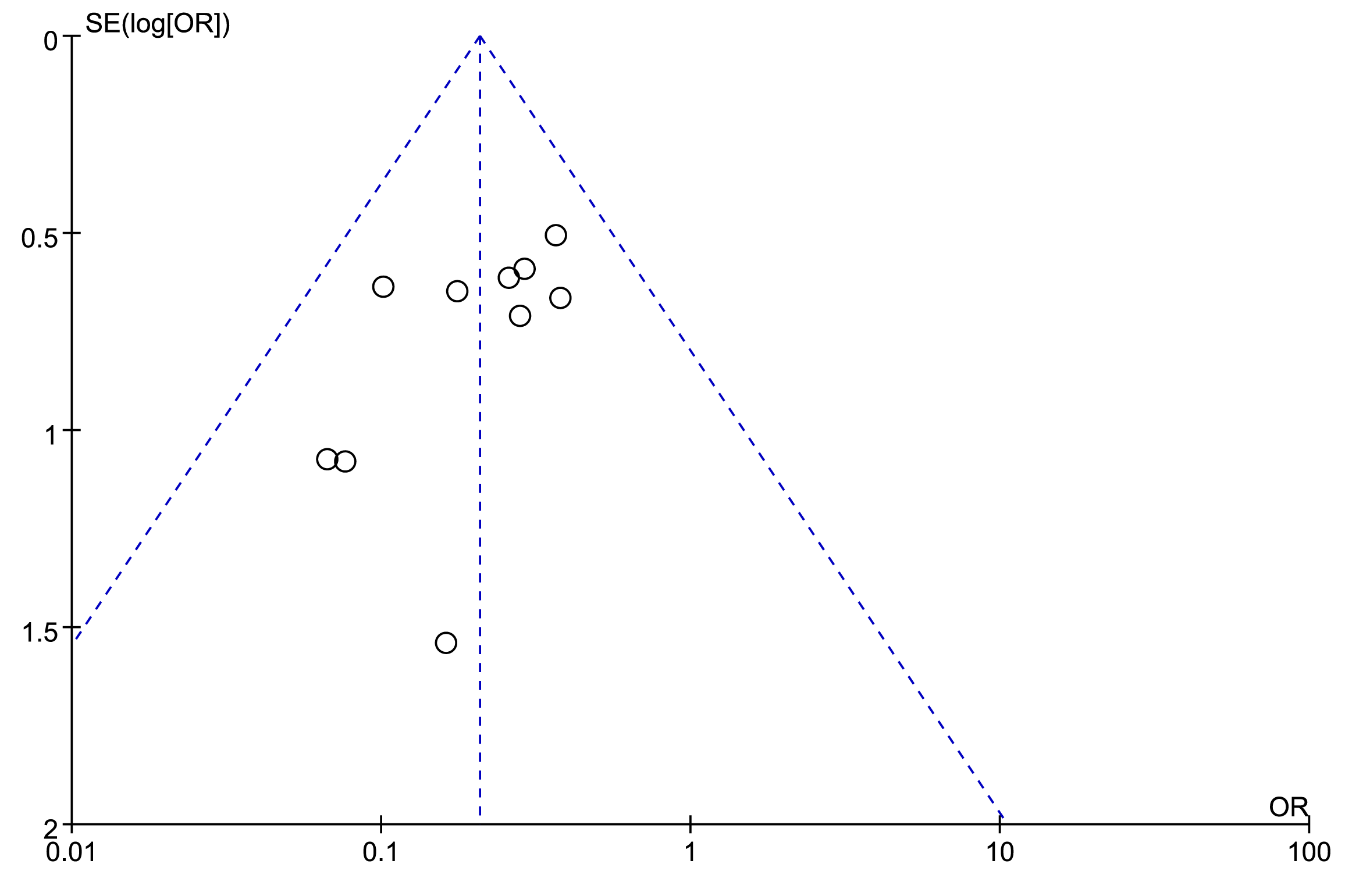

Supplement: Supplementary File 2 — Sensitivity analysis graph: evaluating the stability of the effect of ulinastatin on POCD. [file Image_2.TIF]

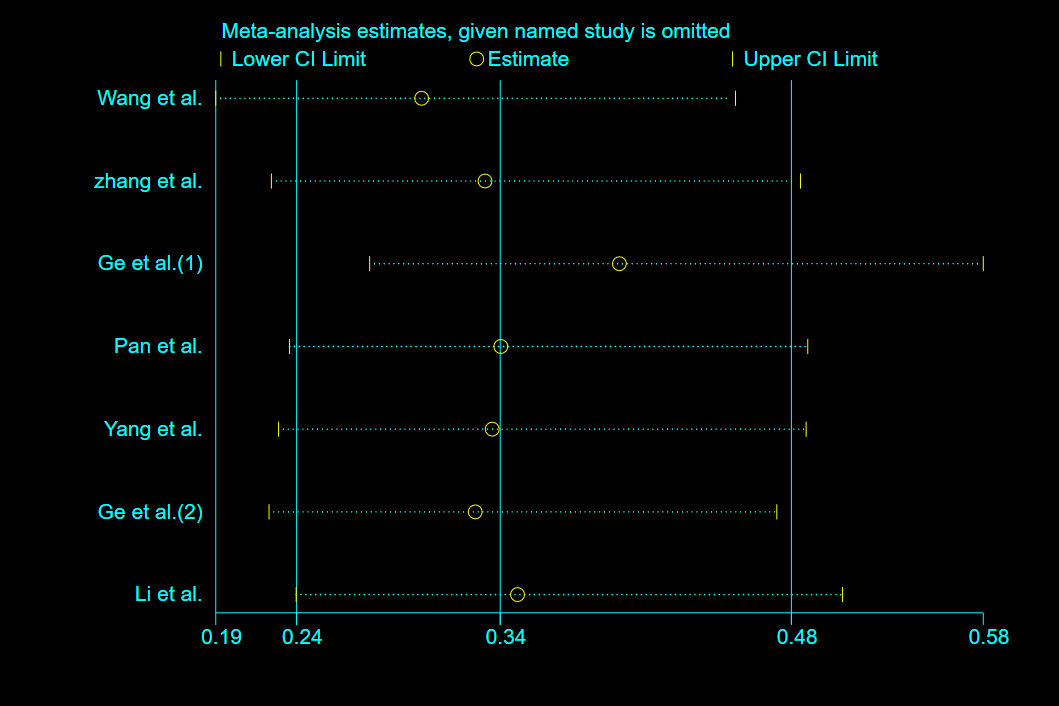

Supplement: Supplementary File 3 — Funnel plot about the incidence of POCD among included studies. [file Image_3.TIF]

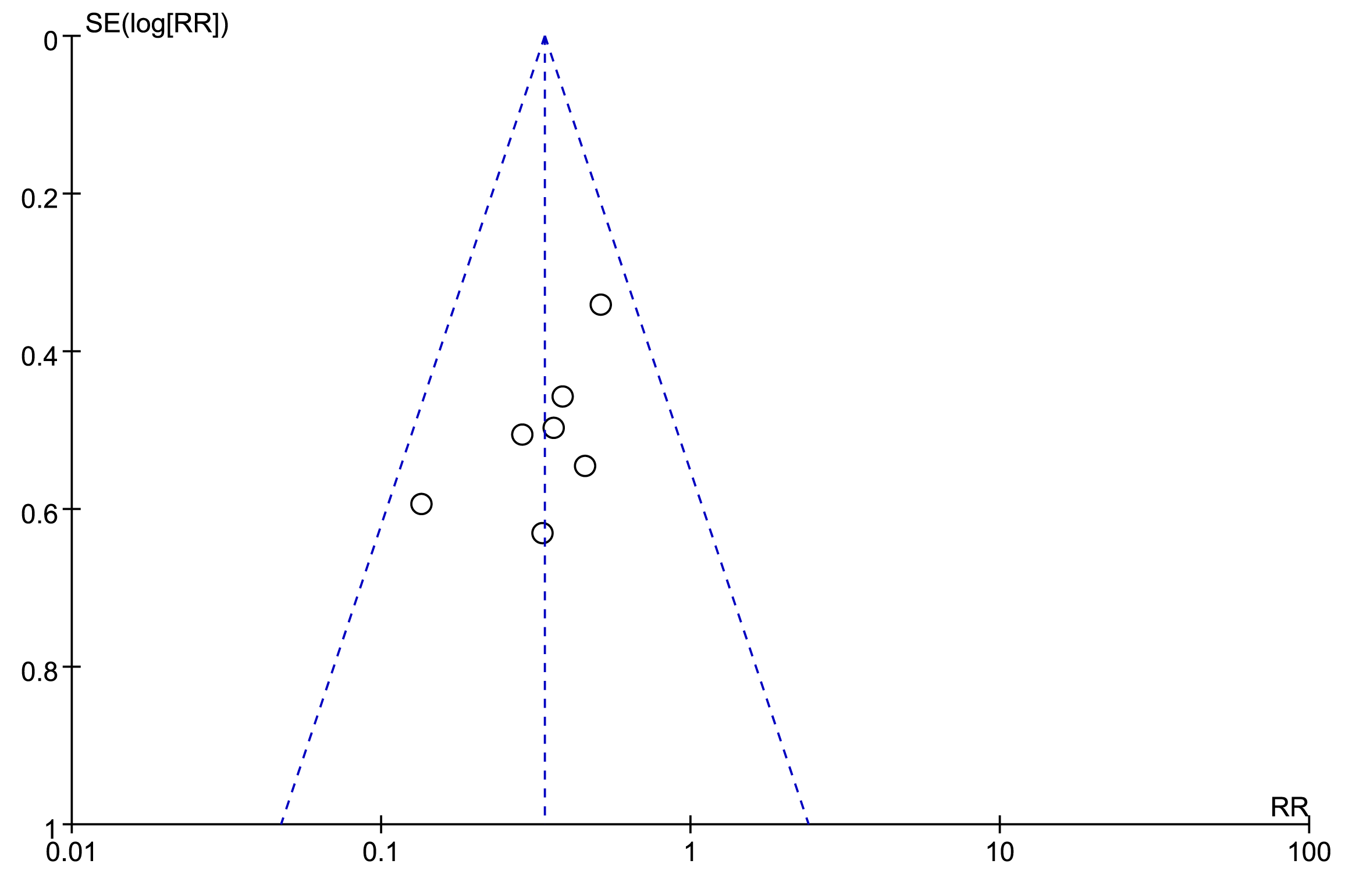

Supplement: Supplementary File 4 — Sensitivity analysis graph: evaluating the stability of the effect of ulinastatin on POCD after removing three articles with publication bias. [file Image_4.TIF]

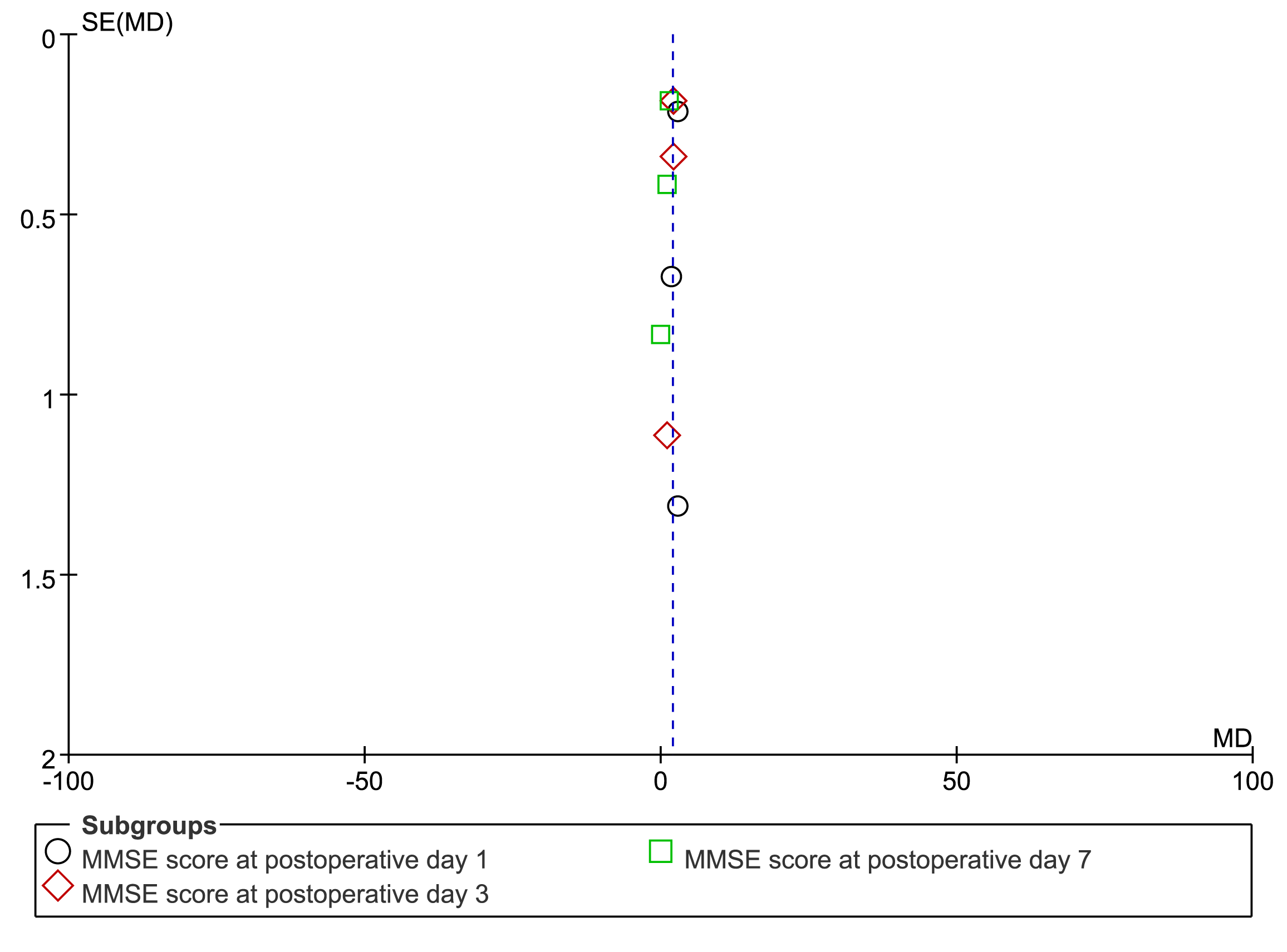

Supplement: Supplementary File 5 — Funnel plot about the incidence of POCD after removing three articles with publication bias. [file Image_5.TIF]
