# Supplementary material for: Effect of Ulinastatin on Early Postoperative Cognitive Dysfunction in Elderly Patients Undergoing Surgery: A Systemic Review and Meta-Analysis
Source: Front Neurosci. 2021 Jun 21;15:618589. doi: 10.3389/fnins.2021.618589 (PMC8265373; doi:10.3389/fnins.2021.618589)
Supplement: Supplementary File 6 — Funnel plot about the MMSE score among included studies. [file Data_Sheet_1.PDF]

Search Strategy:

((("Aged"[Mesh]) OR (Elderly[Title/Abstract])) AND (("urinastatin" [Supplementary Concept]) OR ((((((((((((UTI68[Title/Abstract]) OR (acid-stable protease inhibitor[Title/Abstract])) OR (urinary trypsin inhibitor[Title/Abstract])) OR (urinary trypsin inhibitor (68)[Title/Abstract])) OR (urinary trypsin inhibitor-like inhibitor (43)[Title/Abstract])) OR (UTI(68)[Title/Abstract])) OR (ulinastatin[Title/Abstract])) OR (MR 20[Title/Abstract])) OR (trypsin inhibitor MR-20[Title/Abstract])) OR (MR 20 (magnetic powder) of urinastatin[Title/Abstract])) OR (MR-20 (magnetic powder)[Title/Abstract])) OR (Miraclid[Title/Abstract])))) AND (("Postoperative Cognitive Complications"[Mesh]) OR ((((((((((((((Cognitive Complication, Postoperative[Title/Abstract]) OR (Cognitive Complications, Postoperative[Title/Abstract])) OR (Complication, Postoperative Cognitive[Title/Abstract])) OR (Complications, Postoperative Cognitive[Title/Abstract])) OR (Postoperative Cognitive Complication[Title/Abstract])) OR (Postoperative Cognitive Dysfunction[Title/Abstract])) OR (Cognitive Dysfunction, Postoperative[Title/Abstract])) OR (Cognitive Dysfunctions, Postoperative[Title/Abstract])) OR (Dysfunction, Postoperative Cognitive[Title/Abstract])) OR (Dysfunctions, Postoperative Cognitive[Title/Abstract])) OR (Postoperative Cognitive

Dysfunctions[Title/Abstract])) OR (Postoperative Decline[Title/Abstract])) OR (Decline, Postoperative[Title/Abstract])) OR (Declines, Postoperative[Title/Abstract])) OR (Postoperative Declines[Title/Abstract]))). The search dates ranged from the establishment of each database to February 2020.
