# Supplementary material for: Effect of Ulinastatin on Early Postoperative Cognitive Dysfunction in Elderly Patients Undergoing Surgery: A Systemic Review and Meta-Analysis
Source: Front Neurosci. 2021 Jun 21;15:618589. doi: 10.3389/fnins.2021.618589 (PMC8265373; doi:10.3389/fnins.2021.618589)
Supplement: Supplementary File 7 — Changes in inflammatory factors reported by included studies. [file Data_Sheet_2.pdf]

Study	Time	IL-6		TNF -α		S100β		IL-10	
		U	C	U	C	U	C	U	C
Ge et al.,2015 china(1)	A1	36.1±5.48	34.92±4.68	29.67±4.17	30.84±3.98	0.25±0.03	0.25±0.04	15.24±2.56	14.61±2.45
	B1	38.4±6.34	39.71±6.51	33.12±4.56	33.77±4.38	0.31±0.06	0.31±0.05	17.54±3.35	16.78±4.15
	C1	49.66±5.89	62.9±7.23	37.93±6.80	44.09±11.35	0.77±0.18	0.81±0.23	77.88±16.06	43.36±13.08
	D1	65.14±10.86	90.63±12.06	51.92±6.39	71.26±11.33	0.62±0.16	0.77±0.21	43.28±10.95	32.05±8.21
	E1	48.03±6.01	61.20±6.17	62.55±12.07	80.98±15.33	0.43±0.09	0.47±0.10	24.67±5.01	22.80±3.47
Ge et al.,2015 china(2)	A1	36.1±5.48	34.92±4.68	30.24±4.05	30.84±3.98	0.24±0.05	0.25±0.04	14.84±2.59	14.61±2.45
	B1	37.5±5.62	39.71±6.51	32.52±5.60	33.77±4.38	0.27±0.03	0.31±0.05	16.12±3.56	16.78±4.15
	C1	48.56±6.25	62.9±7.23	38.17±5.70	44.09±11.35	0.75±0.17	0.81±0.23	76.47±17.49	43.36±13.08
	D1	68.16±9.05	90.63±12.06	50.42±3.27	71.26±11.33	0.59±0.18	0.77±0.21	44.48±10.73	32.05±8.21
	E1	47.02±6.73	61.20±6.17	61.30±11.91	80.98±15.33	0.44±0.10	0.47±0.10	22.19±4.05	22.80±3.47
Li et al.,2016 china	A2	2.61±0.33	2.60±0.32	2.41±0.33	2.44±0.41	0.06±0.01	0.06±0.01	19.29±0.13	19.26±0.12
	B2	10.47±2.49	19.37±3.22	4.23±0.67	6.99±0.98	0.12±0.03	0.18±0.03	16.48±2.19	12.39±2.49
	C2	24.38±3.49	45.33±5.39	15.34±2.38	28.21±4.39	0.21±0.03	0.38±0.02	9.81±1.32	8.35±1.32
	D2	18.38±2.93	33.28±3.21	12.37±2.18	19.32±3.21	0.14±0.03	0.24±0.02	14.49±2.21	11.33±1.32
	E2	9.45±2.39	15.38±2.49	6.37±1.32	12.11±2.02	0.07±0.01	0.09±0.02	17.48±4.01	15.34±3.13
Xu et al.,2013 china	A3	7.1 ± 0.1	8.2 ± 0.2	870 ± 490	890 ± 590	0.039 ± 0.012	0.040 ± 0.011	——	——
	B3	55.2 ± 5.1	98.3 ± 4.4	1210 ± 450	1380 ± 860	0.097 ± 0.014	0.129 ± 0.034	——	——
	D3	46.2 ± 4.8	72.2 ± 3.8	1070 ± 540	1190 ± 750	0.086 ± 0.016	0.141 ± 0.029	——	——
	E3	21.4 ± 7.3	45.3 ± 6.3	950 ± 510	960 ± 460	0.057 ± 0.019	0.089 ± 0.038	——	——
	F3	8.2 ± 0.3	9.3 ± 0.4	880 ± 490	890 ± 510	0.042 ± 0.017	0.047 ± 0.018	——	——
Pan et al.,2016 china	A3	454.2±78.2	464.2±115.8	130.4±44.1	135.8±39.3	——	——	——	——
	D3	554.2±89.8	622.2±132.2	155.5±35.9	169.6±44.8	——	——	——	——
	F3	500.5±55.4	600.1±101.5	115.2±19.4	155.1±31.8	——	——	——	——
Wang et al.,2015 china	A3	8.3±1.1	9.1±0.9	——	——	0.057±0.018	0.065±0.022	3.6±0.4	3.8±0.3
	B3	60.7±4.3	100.5±5.0	——	——	0.115±0.024	0.134±0.035	4.5±1.2	4.4±0.9
	D3	57.1±3.7	75.2±4.1	——	——	0.089±0.035	0.110±0.042	5.2±0.8	4.7±0.6
	F3	9.0±0.7	9.5±1.2	——	——	0.062±0.015	0.071±0.017	5.3±0.3	4.7±0.5
Yang et al.,2017 china	A3	451.53±102.4	448±93.72	——	——	0.61±0.04	0.59±0.03	——	——
	D3	511.21±103.24	528.31±93.43	——	——	0.87±0.05	1.06±0.03	——	——
	G3	446.65±96.58	492.74±89.57	——	——	0.67±0.03	0.73±0.05	——	——
Kang et al.,2010 china	A3	——	——	——	——	0.040±0．013	0.041±0．012	——	——
	B3	——	——	——	——	0.095±0．021	0.125±0．031	——	——
	C3	——	——	——	——	0.116±0．017	0.178±0．036	——	——
	D3	——	——	——	——	0.087±0．019	0.142±0．038	——	——
	F3	——	——	——	——	0.043±0．012	0.048±0．015	——	——

Supplementary file 7, Changes in inflammatory factors reported by included studies

Note: Ge et al.,2015 china(1)— large dose of ulinastatin; Ge et al.,2015 china(2)— lower dose of ulinastatin; A1—Pre-operation, B1—Open chest, C1—End of surgery, D1—6h after surgery, E1—24h after surgery; A2—Before CPB, B2—1h after CPB, C2—1h after the end of CPB, D2—4h after surgery, E2—24h after surgery; A3—Pre-operation, B3—end of surgery, C3— 3h after surgery, D3—1 day after surgery, E3—2 day after surgery, F3—3 day after surgery, G3—7 day after surgery.
